# Supersulfides contribute to joint homeostasis and bone regeneration

**DOI:** 10.1016/j.redox.2025.103545

**Published:** 2025-02-11

**Authors:** Miki Maemura, Masanobu Morita, Seiryo Ogata, Yoichi Miyamoto, Tomoaki Ida, Kazuhiro Shibusaka, Soichiro Negishi, Masahiro Hosonuma, Taku Saito, Jun Yoshitake, Tsuyoshi Takata, Tetsuro Matsunaga, Eikan Mishima, Uladzimir Barayeu, Takaaki Akaike, Fumiko Yano

**Affiliations:** a Department of Biochemistry, Graduate School of Dentistry, Showa University, Tokyo, Japan; b Department of Oral and Maxillofacial Surgery, Graduate School of Dentistry, Showa University, Tokyo, Japan; c Department of Environmental Medicine and Molecular Toxicology, Tohoku University Graduate School of Medicine, Sedai, Japan; d Faculty of Arts and Sciences at Fujiyoshida, Showa University, Fujiyoshida, Japan; e Department of Orthodontics, Graduate School of Dentistry, Showa University, Tokyo, Japan; f Department of Pharmacology, Graduate School of Pharmacy, Showa University, Tokyo, Japan; g Sensory & Motor System Medicine, Graduate School of Medicine, The University of Tokyo, Tokyo, Japan; h Center for Integrated Control, Epidemiology and Molecular Pathophysiology of Infectious Diseases, Akita University, Akita, Japan; i Institute of Metabolism and Cell Death, Molecular Targets and Therapeutics Center, Helmholtz Munich, Neuherberg, Germany; j Max-Planck-Institute for Polymer Research, Mainz, Germany

**Keywords:** Supersulfides, Cysteinyl-tRNA synthetase, Osteoarthritis, Bone regeneration, Glutathione trisulfide, Ferroptosis

## Abstract

The physiological functions of supersulfides, inorganic and organic sulfides with sulfur catenation, have been extensively studied. Their synthesis is mainly mediated by mitochondrial cysteinyl-tRNA synthetase (CARS2) that functions as a principal cysteine persulfide synthase. This study aimed to investigate the role of supersulfides in joint homeostasis and bone regeneration. Using *Cars2*^AINK/+^ mutant mice, in which the KIIK motif of CARS2 essential for supersulfide production was replaced with AINK, we evaluated the role of supersulfides in fracture healing and cartilage homeostasis during osteoarthritis (OA). Tibial fracture surgery was performed on the wild-type (*Cars2*^+/+^) and *Cars2*^AINK/+^ mice littermates. Bulk RNA-seq analysis for the osteochondral regeneration in the fracture model showed increased inflammatory markers and reduced osteogenic factors, indicative of impaired bone regeneration, in *Cars2*^AINK/+^ mice. Destabilization of the medial meniscus (DMM) surgery was performed to produce the mouse OA model. Histological analyses with Osteoarthritis Research Society International and synovitis scores revealed accelerated OA progression in *Cars2*^AINK/+^ mice compared with that in *Cars2*^+/+^ mice. To assess the effects of supersulfides on OA progression, glutathione trisulfide (GSSSG) or saline was periodically injected into the mouse knee joints after the DMM surgery. Thus, supersulfides derived from CARS2 and GSSSG exogenously administered significantly inhibited inflammation and lipid peroxidation of the joint cartilage, possibly through suppression of ferroptosis, during OA development. This study represents a significant advancement in understanding anti-inflammatory and anti-oxidant functions of supersulfides in skeletal tissues and may have a clinical relevance for the bone healing and OA therapeutics.

## Highlights

• Supersulfides critically contribute to bone regeneration.
• CARS2-derived supersulfides protect joint cartilage from osteoarthritis.
• Sulfane sulfur of glutathione trisulfide (GSSSG) is incorporated into cells.
• GSSSG treatment ameliorates osteoarthritis progression in mice.
• Supersulfides regulates lipid peroxidation and ferroptosis in osteoarthritis.

## Graphical abstract

**Figure:**
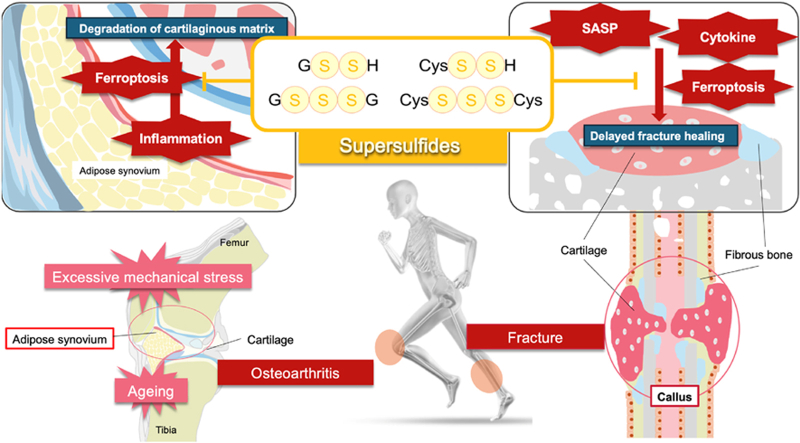

## 1 Introduction

Recent advances in the analytical methodology have revealed the abundance of supersulfides and their function as intrinsic redox regulators in a variety of species, including humans and other mammals [[1], [2], [3]]. Supersulfides are hydropersulfides (RSSH) and polymeric sulfur species with sulfur catenation (RSS_n_R, where n is > 1 and R indicates a hydrogen or an alkyl, or cyclized polysulfides) [1,[4], [5], [6], [7], [8]]. Cysteinyl tRNA synthetase (CARS), especially mitochondrial CARS (CARS2), is regarded as the most important cysteine persulfide synthases (CPERS) in mammals and are responsible for endogenous supersulfide production [1,3,9]. Among the various biological functions of supersulfides [2], their anti-inflammatory actions are important. Treatment with glutathione trisulfide (GSSSG), another supersulfide donor, prevents retinal inflammation in rats [10]. Furthermore, administration of GSSSG ameliorates the pulmonary conditions of these lung diseases as well as that of lungs with influenza virus and SARS-CoV-2 infections [8].

Normal bone fracture healing involves local and transient inflammation, while systemic and excessive inflammation delays fracture healing [[11], [12], [13]]. Excessive oxidative stress not only aggravates physiological bone metabolism but also delays fracture healing [14]. In addition, many studies have indicated the involvement of inflammation and oxidative stress in the pathogenesis and progression of skeletal diseases, including rheumatoid arthritis, periodontitis, osteoporosis, and osteoarthritis (OA) [[15], [16], [17], [18], [19], [20], [21]]. Among various skeletal disorders, OA is highly problematic due to the number of patients [22]. Inflammation and oxidative stress are directly linked to the pathogenesis and exacerbation of OA [21,23]. Because inflammatory processes known to promote lipid peroxidation, subsequently inducing ferroptosis [24], which therefore may contribute to the pathogenesis of bone disorders including fracture healing and OA.

Therefore, we investigated the role of supersulfides in fracture healing and OA by using both wild-type and *Cars2*^AINK/+^ mice, the latter of which possess full tRNA synthetase activity but show impaired CPERS activity [8].

## 2 Materials and methods

### 2.1 Fracture model

To produce fracture model, a transverse osteotomy was conducted at the midpoint of the tibia of *Cars2*^*+/+*^ and *Cars2*^AINK*/+*^ mice, using disk-shaped dental steel bars. The fracture was repositioned, and the full-length of the bone marrow cavity was internally stabilized by inserting a spinal needle. The tibias harvested were analyzed by quantitative reverse transcription-polymerase chain reaction (qRT-PCR) and Bulk RNA-seq. qRT-PCR and Bulk RNA-seq was performed according to previous method [25].

### 2.2 OA experiment

We created a destabilization of the medial meniscus (DMM) model to induce OA in 8-week-old male mice, as previously described [26]. The OA severity was quantified using the Osteoarthritis Research Society International (OARSI) scoring system [26], and synovitis was assessed using a scoring system, as previously described [27]. GSSSG, was administered to the animal via intra-articular injections, followed by assessment of the OA severity at 16 weeks post-surgery.

### 2.3 Supersulfide metabolome analysis of mouse chondrocytes and chondral tissues

HEK293T cells, ATDC5 cells or mouse chondral cells and tissues (joint synovium and cartilage, 5 mg) were homogenized in 0.15 ml or 0.5 ml of cold methanol solution containing 5 mM β-(4-hydroxyphenyl)ethyl iodoacetamide (HPE-IAM) and 50 mM sodium acetate buffer (pH 6.5), after which samples were incubated for 20 min at 37 °C. Following centrifugation (14,000 × g for 10 min at 4 °C), lysate supernatants were diluted with 0.1 % formic acid containing known amounts of isotope-labelled internal standards, and then the LC-ESI-MS/MS (LCMS-8060NX; Shimadzu) measurements were performed. Centrifugation pellets were dissolved in PBS containing 0.1 % SDS, after which protein concentrations were determined by using the BCA assay. LC-ESI-MS/MS conditions and isotope-labelled internal standards synthesis were employed in the same manner as described earlier [1,8].

### 2.4 Cellular uptake analysis using stable isotope labelled GSSSG

HEK293T cells or ATDC5 cells were seeded onto 24-well plate and cultured for overnight. Then the cells were washed three times with DMEM followed by the treatment with various concentrations of GSSG, GSSSG, N-acetylcysteine trisulfide (NAC-S1) or stable isotope labelled GSSSG ([^13^C_2_, ^15^N]GS-[^34^S]-SG[^13^C_2_, ^15^N]) for 3 h at 37 °C. The cells are harvested and subjected to the supersulfide metabolome analysis, as described above.

## 3 Results

### 3.1 Fracture healing was impaired by reduced CPERS activity of CARS2

**Fig. 1:**
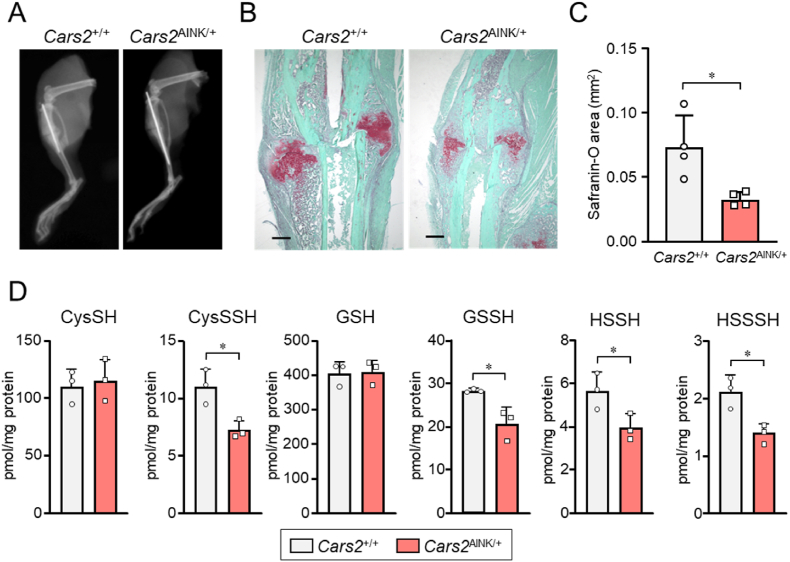
Comparison of bone fracture healing between *Cars2*^*+/+*^ and *Cars2*^AINK*/+*^ mice. (A) Representative soft X-ray images of fracture sites in male *Cars2*^*+/+*^ and *Cars2*^AINK*/+*^ mice at 2 weeks post-fracture. (B) Safranin-O staining of calluses 2 weeks after fracture. Scale bars, 500 μm (C) Semi-quantification of the areas of safranin-O-stained calluses in the tibias of *Cars2*^*+/+*^ and *Cars2*^AINK*/+*^ mice. Data are means ± SD. ∗P < 0.05. Symbols represent individual mice (n = 4 per group). (D) In vivo formation of supersulfides in *Cars2*^*+/+*^ and *Cars2*^AINK*/+*^ mice. Endogenous production of CysSH, GSH, CysSSH, and other related supersulfide metabolites in chondrocytes and chondral tissues obtained from *Cars2*^*+/+*^ and *Cars2*^AINK*/+*^ mice littermates were quantified via LC-MS/MS analysis with HPE-IAM labeling. Data are means ± SD. ∗P < 0.05.

To investigate the role of CPERS activity of CARS2 in endochondral ossification, we examined its involvement in postnatal bone healing under pathological conditions by comparing fracture healing between *Cars2*^+/+^ and *Cars2*^AINK/+^ mice. By using CRISPR-Cas9 genome editing technology, non-synonymous point mutations replaced the pyridoxal-5ʹ-phosphate-binding motif KIIK, which is critical for the CPERS activity of CARS2, with AINK, thereby generating the *Cars2*^AINK^ allele [8]. Since homozygous *Cars2* mutant *Cars2*^AINK/AINK^ mice are embryonic lethal, we used heterozygous *Cars2*^AINK/+^ mice in this study. Fractures were surgically created in the tibias of 12-week-old male mice. Both intramembranous and endochondral ossification occur during fracture healing, and osteochondroprogenitor cells from the periosteum near the fracture sites are the major source of cells that contribute to healing [28,29]. Fracture healing was evaluated 2 weeks post-surgery, when bony bridging at the fracture site is typically observed [28,29]. Soft X-ray analyses revealed impaired callus formation in *Cars2*^+/+^ and *Cars2*^AINK/+^ mice (Fig. 1A). Histological analyses of safranin-O-stained sections were performed to assess differences in healing between the two genotypes (Fig. 1B). *Cars2*^+/+^ mice had large areas of safranin-O-stained soft calluses around the fracture sites, as previously reported [30] (Fig. 1B). However, *Cars2*^AINK/+^ mice had significantly reduced safranin-O-stained soft calluses, compared with *Cars2*^+/+^mice (Figs. 1B and C), suggesting that CPERS activity of CARS2 affects endochondral ossification during fracture healing. The amounts of all supersulfide-related metabolites in chondrocytes and chondral tissues of *Cars2*^AINK/+^ mice were reduced compared with *Cars2*^+/+^mice, as assessed by supersulfide metabolome analysis (Fig. 1D and Fig. S1). Because of haploinsufficiency with the *Cars2*^AINK/+^ mice, the supersulfide production is expected to reduce by 50 % at maximum, which was confirmed by this supersulfide metabolome analysis. These results suggest that a decrease in supersulfides contribute to impaired fracture healing.

### 3.2 Comprehensive mRNA analysis of calluses in the fractures of Cars2^AINK/+^ and Cars2^+/+^ mice

**Fig. 2:**
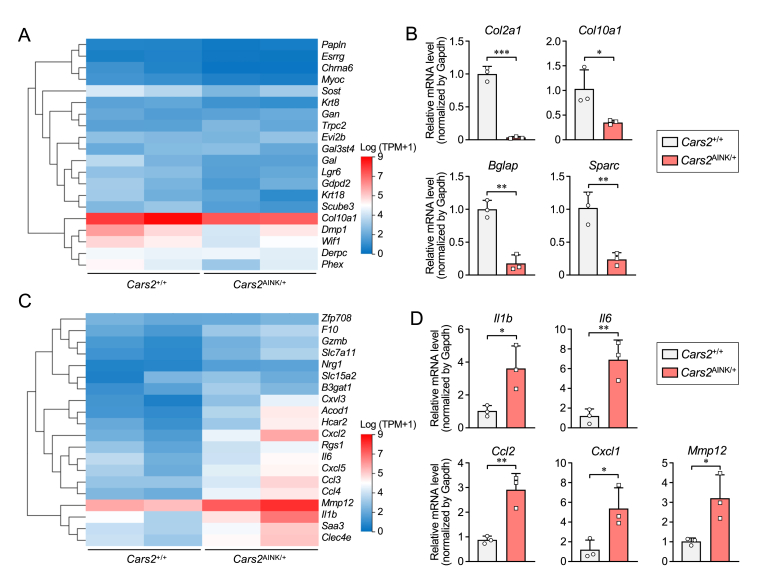
Comprehensive gene expression in the calluses of *Cars2*^*+/+*^ and *Cars2*^AINK*/+*^ mice. Bulk RNA-seq analysis of calluses from the tibias of *Cars2*^*+/+*^ and *Cars2*^AINK*/+*^ mice (n = 2 each) at 2 weeks post-fracture. (A) Heatmap showing the top 20 downregulated differentially expressed genes (DEGs) in calluses of *Cars2*^AINK*/+*^ mice compared to those of *Cars2*^*+/+*^ mice. (B) mRNA levels of markers for endochondral ossification in calluses from the tibias of *Cars2*^*+/+*^ and *Cars2*^AINK*/+*^ mice at 2 weeks post-fracture. (C) The top 20 upregulated DEGs in calluses of *Cars2*^AINK*/+*^ mice, as compared with calluses of *Cars2*^*+/+*^ mice. (D) mRNA levels of inflammatory response markers in calluses from the tibias of *Cars2*^*+/+*^ and *Cars2*^AINK*/+*^ mice at 2 weeks post-fracture. Data are presented as dot plots and means ± SD (n = 3 per group). ∗P < 0.05, ∗∗P < 0.005, ∗∗∗P < 0.0005.

To better understand the gene alterations involved in the bone-healing process in *Cars2*^+/+^ and *Cars2*^AINK/+^ mice, we performed RNA-seq analysis of the calluses of tibias from both genotypes two weeks post-surgery (Fig. 2). Principal component analysis (PCA) and heatmap analyses based on differentially expressed genes (DEGs) revealed distinct gene expression patterns between the two genotypes (n = 2 per group) (Figs. S2A and B). Heatmaps displayed the top 20 downregulated genes in the calluses of *Cars2*^AINK/+^ compared with that of *Cars2*^+/+^ mice (Fig. 2A). Osteogenic factors such as *Col10a1*, *Dmp1*, and *Sost* were significantly decreased in *Cars2*^AINK/+^ mice, as compared with those in *Cars2*^+/+^ mice. To confirm the gene alterations observed in the RNA-seq analysis, qRT-PCR was used to investigate the mRNA expression in the calluses of *Cars2*^AINK/+^ and *Cars2*^+/+^ mice (n = 3 per group). Besides the aforementioned genes, other endochondral ossification markers, *Col2a1*, *Bglap*, and *Sparc* expression levels were also significantly reduced in *Cars2*^AINK/+^ mice (Fig. 2B).

Heatmaps displayed the top 20 upregulated genes in the calluses of *Cars2*^AINK/+^ compared with that of *Cars2*^+/+^ mice (Fig. 2C). Notably, inflammatory response markers such as *Il1b*, *Il6*, and *Mmp12* were significantly increased in *Cars2*^AINK/+^ mice. Inflammation- and immune-related markers, which were recently identified as senescence-associated secretory phenotype (SASP) markers, together with above genes, such as *Ccl2* and *Cxcl1* were also upregulated in *Cars2*^AINK/*+*^ mice, as compared to those in *Cars2*^*+/+*^ mice (Fig. 2D).

Next, we compared biological terms and pathways based on DEGs in the calluses of *Cars2*^AINK/+^ and *Cars2*^+/+^ mice (Fig. S2C). According to ingenuity pathway analysis (IPA), inflammation- and immune-related pathways were upregulated, while mesenchymal differentiation and anabolic pathways were downregulated in the calluses of *Cars2*^AINK/+^ mice compared with those in the calluses of *Cars2*^+/+^ mice (Fig. S2C). The top 20 down- and upregulated pathways, as determined by their |z-score| values, are shown in Supplemental Tables S2 and S3. Notably, these RNA-seq and IPA results aligned with previous findings [8], including the critical role of CARS2 in suppressing inflammatory responses.

These alterations in gene expression and pathways suggest that CARS2*,* which produces supersulfides, may regulate endochondral ossification accompanied by suppressing inflammatory responses and specifically targeting SASP markers.

### 3.3 Accelerated OA development in *Cars2*^AINK/+^ mice

**Fig. 3:**
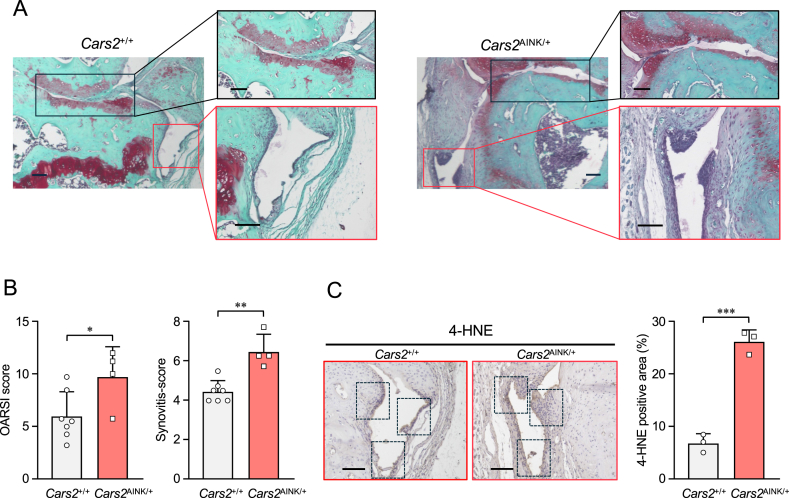
Development of osteoarthritis (OA) in *Cars2*^*+/+*^ and *Cars2*^AINK*/+*^ mice. (A) At 16 weeks post-DMM surgery, knee joints were stained with safranin-O (left panels). Boxed areas in the left panel are shown at higher magnification, articular cartilage (upper right panels, outlined in black), and synovial lesions (lower right panels, outlined in red). Representative images are shown. Scale bars, 100 μm. (B) Quantification of OA development using Osteoarthritis Research Society International (OARSI) histologic scoring for *Cars2*^*+/+*^ [n = 7] and *Cars2*^AINK*/+*^ [n = 4] mice. Severity of synovitis assessed using synovitis scores (*Cars2*^*+/+*^ [n = 7] and *Cars2*^AINK*/+*^ [n = 4] mice). Data are means ± SD. ∗P < 0.05, ∗∗P < 0.005. (C) Immunohistochemical assessment of 4-hydroxy-2-nonenal (4-HNE) expression in the synovium of knee joints that correspond to the boxed area in (A). The rates of 4-HNE-positive areas in the boxes are shown in the panels in right panel. Data are means ± SD. ∗∗∗P < 0.0005.

*Cars2*^AINK/+^ mice were also used to investigate the role of CARS2 in the pathological development of OA. Although *Cars2*^AINK/+^ mice did not show obvious impairments in skeletal growth or body weight (Figs. S3A and B), we employed the DMM model [26] using 12-week-old *Cars2*^AINK/*+*^ and *Cars2*^*+/+*^ mice to assess the specific role of CARS2 in knee joint homeostasis (Fig. 3). Safranin-O staining and OARSI scores indicated that OA progression was significantly accelerated in *Cars2*^AINK/+^ mice (Figs. 3A and B). In contrast, the sham-operated knee joints of *Cars2*^AINK/*+*^ and *Cars2*^*+/+*^ mice did not exhibit OA cartilage changes (Figs. S4A and B). Cartilage degeneration observed in *Cars2*^AINK/+^ mice prompted us to evaluate histological changes in the synovium and assess the severity of synovitis. To this end, synovitis scores were measured 16 weeks post-surgery, even though the condition may have been chronic at that time [31]. *Cars2*^AINK/*+*^mice showed higher synovitis scores than *Cars2*^*+/+*^ mice (Fig. 3B). We next focused on the generation of 4-hydroxy-2-nonenal (4-HNE), a downstream product and indicator of lipid peroxidation, in the synovium of knee joints [24,32]. Immunohistochemical analysis using an anti-4-HNE antibody confirmed that 4-HNE generation was significantly higher in the OA synovium of *Cars2*^AINK/+^ mice compared to that of *Cars2*^+/+^ mice and was accompanied by an increased synovitis score (Fig. 3C). In contrast, 4-HNE generation was not detected in the sham-operated knee joints of either *Cars2*^AINK/*+*^ and *Cars2*^*+/+*^ mice (Fig. S4C). These data indicate that CARS-derived supersulfides play a protective role in preventing cartilage degeneration and synovial inflammation and ultimately slow OA progression by mitigating oxidative stress.

### 3.4 Suppression of OA progression by GSSSG administration

**Fig. 4:**
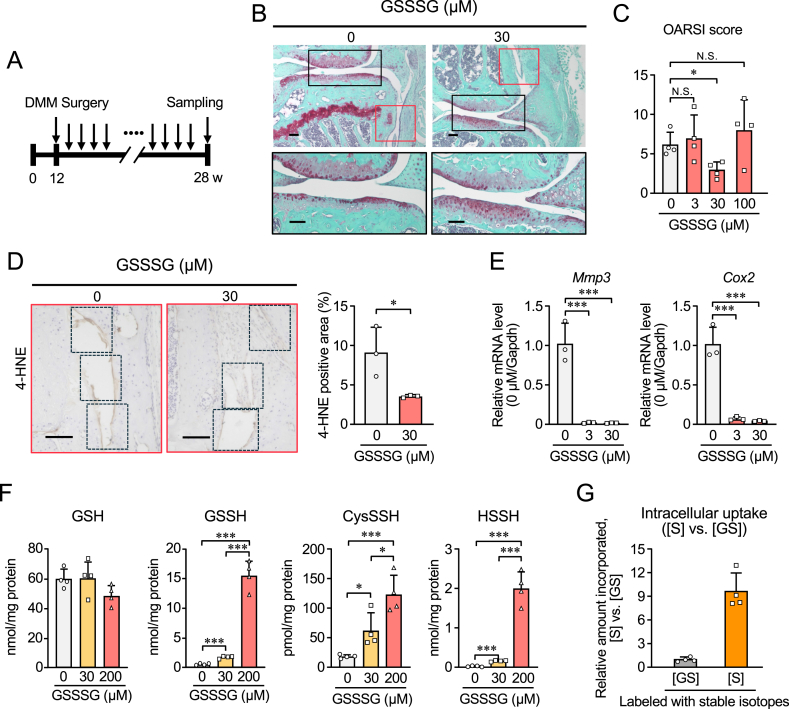
Effects of intra-articular administration of GSSSG on mouse OA model induced surgically and intracellular uptake of supersulfides in ATDC5 cells. (A) A scheme of GSSSG administration to the OA model used in this study. The intra-articular injections were initiated at 12 weeks post-DMM surgery and repeated 16 times once a week for consecutive weeks. (B) At 16 weeks post-surgery and GSSSG treatment, knee joints were stained with safranin-O. Areas within the articular cartilage outlined in black in the upper panels are shown in the lower panels. Scale bar, 100 μm. (C) Semi-quantification of OA development using OARSI histologic scoring in the GSSSG-treated OA model. Data are means ± SD. ∗P < 0.05 vs. 0 μM; N.S., not significant. Each group (0, 3, 30, 100 μM treatment) includes 4 mice. (D) Immunohistochemical assessment of 4-HNE formation in the synovium of knee joints of GSSSG-treated mice at 16 weeks after OA induction. High-magnification images of the areas outlined in red in (B) are shown. The two left panels show representative images; the right panel indicates the rates of 4-HNE-positive areas in the box regions. Data are means ± SD. ∗P < 0.05, vs. 0 μM; Student's t-test. (E) mRNA levels of inflammatory markers (*Mmp3* and *Cox2*) in mouse ASF treated with different concentrations of GSSSG (0, 3, 30 μM) and exposed to 1 ng/ml IL-1β for 24 h Data are means ± SD (n = 3). ∗∗∗P < 0.0005. (F) Supersulfide metabolome analysis with ATDC5 cells treated with various doses of GSSSG (0, 30, 200 μM) for 3 h. Data are means ± SD (n = 4). ∗P < 0.05, ∗∗∗P < 0.001. (G) Intracellular uptake of stable isotope-labelled GS ([GS]) and S ([S]) with ATDC5 cells treated with stable isotope-labelled GSSSG ([^13^C_2_, ^15^N]GS-[^34^S]-SG[^13^C_2_, ^15^N], 200 μM) for 3 h. Amounts of intracellular [GS] and [S] were quantified (Fig. S9A) and their relative ratios ([S] vs. [GS]) are shown. Data are means ± SD (n = 4).

To further elucidate the role of GSSSG in OA, we next investigated its effects in a surgically induced mouse model. GSSSG (3, 30, and 100 μM) or saline (vehicle control) were injected into the knee joints of mice once a week for 16 weeks post-surgery (Fig. 4A). Safranin-O staining and OARSI scores demonstrated that the OA progression was significantly suppressed in the 30 μM GSSSG group compared with the vehicle group (Figs. 4B, C and Figs. S5A and B). However, this protective effect was not observed in the 3 or 100 μM GSSSG group (Fig. 4C and Fig. S5A). Additionally, treatment with 30 μM GSSSG led to a reduction in 4-HNE generation within the OA synovium (Fig. 4D and Fig. S5C). These data indicate that GSSSG may be a disease-modifying OA drug that is capable of preventing the cartilage degeneration and synovial inflammation that contribute to OA progression.

### 3.5 Effects of GSSSG on primary ASFs

We also examined the effect of GSSSG on primary adipose synovial fibroblasts (ASFs) from murine knee joints, because the synovium is crucial in maintaining knee joint homeostasis and contributing to OA pathogenesis [31,33]. To simulate the *in vitro* hypoxic conditions of knee joints, we cultured primary mouse ASFs under hypoxic conditions (2 % O_2_, 5 % CO_2_) and investigated the effects of GSSSG under IL-1β-induced inflammatory conditions, which are commonly used to model synovial inflammation *in vitro* [18]. Mouse ASFs cultured in conditions without the addition IL-1β exhibited a spindle-like shape, which is characteristic of highly proliferative ASFs cells under hypoxic conditions (Fig. S5D). However, 24-h treatment with IL-1β changed the ASF morphology to a round shape, even under hypoxic conditions. Notably, some mouse ASFs reverted to a spindle-like shape when treated with 30 μM GSSSG during IL-1β stimulation (Fig. S5D).

To confirm these morphological changes of the ASFs, we performed qRT-PCR to investigate mRNA expression in mouse ASF. GSSSG treatment, particularly 30 μM, suppressed the IL-1β-induced expression of the inflammation markers *Mmp3* and *Cox2* in mouse ASFs (Fig. 4E). Along with the result, the inflammation markers were increased in *Cars2*^AINK/*+*^ mice, which suggests that supersulfides inhibit the expression of inflammation markers. Taken together, these data suggest that GSSSG helps suppress the inflammation in synovial cells from knee joints under hypoxic conditions.

We also investigated whether the addition of GSSSG indeed increases intracellular supersulfides levels. We treated HEK293T cells with GSSG, GSSSG, or NAC-S1. No increase in supersulfides was observed with GSSG treatment. In contrast, supersulfide levels were significantly elevated following treatment with GSSSG or NAC-S1 (Fig. S6). Subsequently, chondrogenic ATDC5 cells were treated with various concentrations of GSSSG, and intracellular supersulfide metabolome analysis demonstrated a dose-dependent increase in supersulfide levels (Fig. 4F and Fig. S8). The observed increase of supersulfides following GSSSG or NAC-S1 treatment suggests that the trisulfide structure of these compounds having completely different side chain structures may play an important role in uptake into cells. The intracellular GSSH was remarkably elevated up to a mM concentration (1.23 mM) that is comparable to that of GSH (3.86 mM) in ATDC5 cells after treatment with GSSSG, which was much greater than that in GSSSG treated HEK293T cells (Fig. 4F, Figs. S6 and S8). Intriguingly, the magnitude of decrease in the amount of GSH in ATDC5 cells was found to be almost consistent to that of the increase in GSSH for the same cells after treatment with GSSSG (Fig. 4F and Fig. S10).

Then, we conducted the cellular uptake analysis with GSSSG labelled with stable isotopes. Following the cellular uptake analysis with GSSSG labelled with stable isotopes, [^13^C_2_,^15^N]GS-[^34^S]-SG [^13^C_2_,^15^N], the primary stable isotope–labelled GSSH and GSSSH species detected intracellularly were identified as GS-[^34^S]-H and GS-[^34^S_2_]-H, respectively (Fig. S7A and Fig. S9A). Furthermore, the [^13^C_2_,^15^N]GS -labelled species were present at approximately one-tenth the level of the ^34^S-containing molecule species (Fig. 4G and Fig. S7B, S9B). The findings suggest that only the sulfane sulfur [S] of GSSSG undergoes intracellular uptake.

## 4 Discussion

Herein we found that supersulfides are indispensable for the progression of fracture healing in a surgical mouse model. Furthermore, our discovery that suppressed production of supersulfides deteriorated OA, while intra-articular administration of a supersulfide donor inhibited cartilage degeneration, underscores the essential role of supersulfides in maintaining joint homeostasis. This study also highlights the necessity of supersulfide suppression of inflammation for these skeletal functions. These analyses were facilitated by the generation of *Cars2*^AINK/*+*^ mice, a novel model characterized by specific impairment in CPERS [8]; although reduced supersulfids were detected in *Cars2*^AINK/*+*^ mice, tRNA synthetase activity remained unaffected.

After bleeding and inflammatory responses occur at the fracture site, the cartilage forms a soft callus that is then replaced by bone tissue [34]. Senescent cells secrete various factors, including inflammatory cytokines, chemokines, and growth factors, that are involved in age-related changes, a phenomenon known as SASP [35]. The fraction of cells expressing senescent phenotypes increases in calluses at fracture sites, and SASP is involved in delayed fracture healing [36]. The impaired production of supersulfides in *Cars2*^AINK*/+*^ mice may accelerate the cellular senescence in the callus, resulting in delayed fracture healing that is passively mediated, at least partially, by SASP.

*CARS2* expression decreases when human THP-1 macrophages differentiate into proinflammatory M1 macrophages, whereas *CARS2* increases when they differentiate into anti-inflammatory M2 macrophages [37]. Our IPA study on the role of CARS2 in the endochondral ossification suggested that *Cars2*^AINK/+^ mice showed upregulation of the pathogen-induced cytokine storm signaling pathway, potentially associated with altered supersulfide levels [8]. From this data analysis, it is reasonable to assume that supersulfides suppress inflammatory and immunological responses in the skeletal system.

Previous studies, including ours, have suggested that degeneration of the synovium precedes that of cartilage in OA pathogenesis [31,33]. Various factors, such as inflammatory cytokines, chemokines, and metalloproteinases are essential in the progression from synovial to cartilage degeneration [31,33]. In this study, we found the regeneration of the joint synovium was impaired in the OA model of *Cars2*^AINK/+^ mice produced by the DMM surgery, in which lipid peroxidation was accelerated as evidenced by increased 4-HNE formation in the *Cars2*^AINK/+^ OA model (Fig. 3). This result clarified the potential cytoprotective function of supersulfides against lipid peroxidation. The beneficial effect of supersulfide was supported by observation that GSSSG treatment suppressed the cartilage degeneration and lipid peroxidation in the same OA model (Fig. 4). In fact, GSSSG has demonstrated protective effects against retinal inflammation [10], neurodegeneration in the spinal cord [38], pulmonary degenerations in SARS-CoV-2 infection [8]. It is also suggested that supersulfides suppress ferroptosis induced by lipid peroxidation, highlighting their crucial role in cellular protection and joint homeostasis [39].

In addition to direct tissue protection from lipid peroxidation by supersulfides, the maintenance of energy production through sulfur respiration may contribute to beneficial effects of supersulfides in the OA pathogenesis [1,40]. Mitochondrial dysfunction has been implicated in OA models [41,42]. Our previous studies revealed reduced supersulfides production and mitochondrial dysfunction in *Cars2*-deficient (*Cars2*^+/−^) and AINK mutant (*Cars2*^AINK/+^) mice, highlighting a link between CARS2-derived supersulfides and energy metabolism [1,8]. This suggests the impaired mitochondrial function in synovial and cartilage tissues of *Cars2*^AINK/+^ mice exacerbates OA pathology; though further investigation is needed to fully elucidate the mitochondria-dependent mechanisms.

The phenotypic changes of *Cars2*^+/−^ and *Cars2*^AINK/+^ can be manifested and explained by the reduction of superuslfides such as CysSSH, GSSH, HSSH, and HSSSH in Fig. 1, as was reported in our recent paper [8]. Specifically, it is already known that, during acute and subacute oxidative and inflammatory stresses, the reactive hydropersulfides and hydropolysulfides are more susceptible for various oxidants and therefore more quickly consumed than their parental thiols like CysSH and GSH, as we recently clarified in *Cars2*^AINK/+^ mice [8]. Such a remarkable chemical and biochemical trend of nucleophilicity (i.e., anti-oxidant activities) and thereby strong cytoprotection mediated by reactive supersulfides are verified and supported by a number of earlier findings including ours, which show their potent anti-lipid peroxidation and anti-ferroptotic effects [2,39]. In fact, *Cars2* mutants (*Cars2*^+/−^ and *Cars2*^AINK/+^) mostly show 50 % reduction of supersulfides without affecting parental CysSH and GSH, as we confirmed in earlier [8] and the present work (Fig. 1 and Fig. S1). All these data thus logically indicate that impaired supersulfide production in *Cars2*^AINK/+^ mice should cause higher damage by the oxidative stress than that in the *Cars2*^+/+^ mice.

Our study currently conducted showed very effective incorporation of a sulfane sulfur component [S] of GSSSG (rather than the glutathione moiety) into chondrocytes, possibly via a specific pathway for the cellular uptake, of which exact mechanism still remains unclear. The [S] incorporation appears to lead to the intracellular formation of various supersulfides including GSSH, along with other [S]-related derivatives such as various hydropersulfides. Therefore, the pharmacological and cytoprotective consequences of GSSSG reported herein may be caused by the direct anti-lipid peroxidation and anti-ferroptotic effects of [S]-containing supersuflide derivatives, rather than through the GSH-dependent mechanism. To further support this notion, we identified that mouse chondrogenic ATDC5 cells showed tremendous accumulation of GSSH following GSSSG treatment (Fig. 4F). The baseline concentrations of GSSH formed in HEK293T and ATDC5 cells are 4.0 μM and 44.1 μM, respectively as we determined in this study. These values were elevated remarkably; for example, in ATDC5 cells, it reached particularly high levels over 1 mM range (up to 1.23 mM), after 200 μM GSSSG treatment (Figs. S6 and S8). Besides, in case of the in vivo OA study, to the animals were administered the multiple intra-articular injections of GSSSG (a single dose of 100 μM/week, 16 times for 16 weeks), which is expected to induce extremely high concentrations of GSSH and its related reactive supersufldes in the cartilage of mice. These conditions which result in the excessive amount of GSSH may potentially lead to reductive cytotoxicity in this OA model. In fact, such an apparently opposite consequence may provide a plausible explanation for the present observation of failure in attenuating OA progression by a high dose (100 μM) GSSSG treatment (Fig. 4C).

This study may have some limitation, however. For example, we sought to minimize the experimental variability as much as possible by using only male mice; although the disease status is known to be influenced by gender-specific factors such as estrogen and body weight among OA patients. These factors that differ between the genders may differently affect the OA progression in the human subjects than in the mouse model used in this study.

## 5 Conclusions

Our study is the first to clarify the beneficial role of supersulfides in physiology and pathophysiology of the skeletal system. Our results not only highlight the possibility of using supersulfide donors for the prevention and treatment of various skeletal diseases but also suggest their potential for the development of therapeutic agents for a wide range of age-related and inflammatory diseases. The anti-inflammatory and anti-oxidant effects of supersulfides in skeletal tissues may have far-reaching implications for the field of medicine and may open up a new avenue for the development of various disease therapeutics.

## CRediT authorship contribution statement

**Miki Maemura:** Writing – review & editing, Writing – original draft, Visualization, Methodology, Investigation, Formal analysis, Data curation, Conceptualization. **Masanobu Morita:** Writing – original draft, Validation, Supervision, Resources, Methodology, Conceptualization. **Seiryo Ogata:** Writing – review & editing, Visualization, Methodology, Investigation, Formal analysis, Data curation, Conceptualization. **Yoichi Miyamoto:** Writing – review & editing, Writing – original draft, Visualization, Supervision, Resources, Project administration, Methodology, Investigation, Formal analysis. **Tomoaki Ida:** Data curation, Methodology. **Kazuhiro Shibusaka:** Investigation, Formal analysis. **Soichiro Negishi:** Investigation, Formal analysis. **Masahiro Hosonuma:** Visualization, Methodology, Investigation, Data curation. **Taku Saito:** Supervision, Resources, Methodology, Conceptualization. **Jun Yoshitake:** Resources, Methodology. **Tsuyoshi Takata:** Supervision, Resources, Methodology, Conceptualization. **Tetsuro Matsunaga:** Visualization, Supervision, Resources, Methodology, Conceptualization. **Eikan Mishima:** Supervision, Resources, Project administration, Methodology, Conceptualization. **Uladzimir Barayeu:** Supervision, Resources, Project administration, Methodology, Conceptualization. **Takaaki Akaike:** Writing – review & editing, Writing – original draft, Validation, Supervision, Resources, Project administration, Methodology, Funding acquisition, Conceptualization. **Fumiko Yano:** Writing – review & editing, Writing – original draft, Validation, Supervision, Resources, Project administration, Methodology, Investigation, Funding acquisition, Formal analysis, Data curation, Conceptualization.

## Funding sources

This work was supported in part by Transformative Research Areas, International Leading Research, Scientific Research [(S), (B), (C), Challenging Exploratory Research] from the Ministry of Education, Culture, Sports, Science and Technology (MEXT), Japan, to T. A. (18H05277, 21H05258, 21H05263, 22K19397 23K20040 and 24H00063), F.Y. (23K27796), Y.M. (24K02649), T. M. (22K06893), S. O. (23K14333), T.T. (23K06094) and M. Morita. (23K06145); by a Japan Society for the Promotion of Science (JSPS) fellowship to U. B. (PE23749); by Japan Science and Technology Agency, Japan, CREST Grant Number JPMJCR2024, to T. A.; and by a grant from the Japan Agency for Medical Research and Development (AMED) to T. A. (JP21zf0127001) and the Nakatomi Foundation (to M. Maemura).

## Declaration of competing interest

The authors declare that they have no competing interests.

## Data Availability

The raw and processed data used in this study have been deposited in GEO under accession number GSE280156 and are publicly available as of the date of publication.

## Abbreviations

4-HNE: 4-hydroxy-2-nonenal
ASF: adipose synovial fibroblasts
CARS: cysteinyl tRNA synthetase
CARS2: mitochondrial cysteinyl tRNA synthetase
CysSSH: cysteine hydropersulfide
DEGs: differentially expressed genes
DMEM: Dulbecco's modified Eagle's medium
ETC: electron transfer chain
Gapdh: glyceraldehyde-3-phosphate dehydrogenase
GSSSG: glutathione trisulfide
IACUC: Institutional Animal Care and Use Committee
IL: interleukin
IPA: ingenuity pathway analysis
LPS: lipopolysaccharide
NAC-S1: *N*-acetyl cysteine trisulfide
OA: osteoarthritis
PBS: phosphate-buffered saline
PCA: principal component analysis
qT-PCR: quantitative reverse transcription-polymerase chain reaction
RIN: RNA integrity number
ROS: reactive oxygen species
RSSH: hydropersulfides
SASP: senescence-associated secretory phenotype
SD: standard deviation
