# Supplementary material for: Supersulfides contribute to joint homeostasis and bone regeneration
Source: Redox Biol. 2025 Feb 11;81:103545. doi: 10.1016/j.redox.2025.103545 (PMC11893308; doi:10.1016/j.redox.2025.103545)
Supplement: Multimedia component 1 [file mmc1.pdf]

Supplementary Information

Supersulfides contribute to joint homeostasis and bone regeneration

Miki Maemura[a, b,1], Masanobu Morita[c,1], Seiryo Ogata[c,1], Yoichi Miyamoto[d, 1], Tomoaki Ida[c], Kazuhiro Shibusaka[a, e], Soichiro Negishi[a, b], Masahiro Hosonuma[f], Taku Saito[g], Jun Yoshitake[c], Tsuyoshi Takata[c], Tetsuro Matsunaga[c,h], Eikan Mishima[i], Uladzimir Barayeu[j], Takaaki Akaike[c *], Fumiko Yano[a *]

*Correspondence should be addressed to: Takaaki Akaike, M.D., Ph.D [1] and Fumiko Yano, D.D.S., Ph.D [2].

1. Tohoku University Graduate School of Medicine
Department of Environmental Medicine and Molecular Toxicology
2-1, Seiryo-machi, Aoba-ku, Sendai, 980-8575, JAPAN
Tel:(＋81)-22-717-8164
E-mail: takaike@med.tohoku.ac.jp

2. Showa University
Department of Biochemistry, School of Dentistry, Showa University, 1-5-8 Hatanodai, Shinagawa-ku, Tokyo, 142-0064, JAPAN
Tel: (+81)-3-3784-8163
E-mail: fumikoyano@dent.showa-u.ac.jp

This file includes:

1. Supplemental Methods
2. Supplemental Figure 1-10
3. Supplemental Table 1-3

1. Supplemental Methods

1.1. Animal experiments

1.1.1. Animals

All animal experiments were conducted in accordance with the 2011 *Guide for the Use and Care of Laboratory Animals* guidelines from the Institute for Laboratory Animal Research. The animal experiments were approved by the Institutional Animal Care and Use Committee (IACUC) of Showa University (Approval No. 224037). All animals were housed in a facility supervised by the IACUC, maintained at 18– 22°C with a 12-h light/dark cycle, and provided with free access to food and water. In each experiment, we compared the genotypes of littermates maintained on a C57BL/6J background. *Cars2*$^{\mathrm{AINK/+}}$ mutant mice, the KIIK motif of CARS2, which is required for supersulfide production, was heterozygously replaced with AINK, as previously described [8]. *Cars2*$^{\mathrm{AINK/+}}$ mice, which retain full tRNA synthetase activity but show impaired cysteine persulfide synthase (CPERS) activity. Due to haploinsufficiency in the *Cars2*$^{\mathrm{AINK/+}}$ mice, the supersulfide production is expected to reduce by 50% at maximum. *Cars2*$^{+/+}$ mice and *Cars2*$^{\mathrm{AINK/+}}$ littermates were used for the fracture and OA models, with *Cars2*$^{+/+}$ mice serving as controls. Mice were monitored post-operatively using a hot mat for warmth, and we performed a 24-hour observation period. All procedures were conducted with care and in accordance with ethical guidelines to minimize any discomfort.

1.1.2. Fracture model

Eight 12-week-old male mice per group were administered general anesthesia with isoflurane in O_2, and the left hind limbs were sterilized for surgery. A 15-mm

longitudinal incision was made, and the muscle underwent blunt dissection to expose the tibia, as previously described [2]. A transverse osteotomy was created at the midpoint of the tibia using disk-shaped dental steel bars. The fracture was repositioned, and the full-length of the bone marrow cavity was internally stabilized by inserting a 23 G spinal needle (SN-2370, Terumo Clinical Supply, Kakamigahara, Japan). After saline irrigation, the skin was sutured with 4-0 nylon stitches. Fourteen days after surgery, the mice were euthanized, and the tibias that contain osteochondroprogenitor cells were harvested.

1.1.3. OA model

We created a destabilization of the medial meniscus (DMM) model to induce OA in 8-week-old male mice, as previously described [3]. A sham operation was performed on the contralateral knee joint using the same approach but without destabilization of the medial meniscus. All surgical procedures were conducted under general anesthesia using a surgical microscope. At 16 weeks post-surgery, mice were assessed for DMM modeling. All mice were maintained under identical conditions, with a maximum of five mice per cage. The OA severity was quantified using the Osteoarthritis Research Society International (OARSI) scoring system [3] , and synovitis was assessed using a scoring system, as previously described [4]. Both assessments were performed by five observers who were blinded to the experimental groups.

1.1.4. Intra-articular injections

For the intra-articular injections, 0, 3, 30 and 100 μM of GSSSG [1,5] were dissolved in saline (sterile 0.9% NaCl; Otsuka Pharmaceutical Factory, Naruto, Japan) and adjusted

to a pH of 7.8. To determine the effective doses of GSSSG, we performed OA surgery on C57BL/6J male mice (4 per group) and administered intra-articular injections (10 μL) of 0, 3, 30 or 100 μM GSSSG, once a week, for 16 weeks post-surgery. The OA severity was assessed at 16 weeks post-surgery.

1.2. Histological analyses

Tissue samples were fixed overnight in 4% paraformaldehyde/phosphate-buffered saline at 4°C. The samples were then decalcified in 10% ethylenediaminetetraacetic acid (EDTA; pH 7.4, NACALAI TESQUE, Kyoto, Japan) at 4°C for 4 weeks and embedded in paraffin; 4-μm thick coronal sections were cut from the paraffin blocks. Safranin-O staining was performed, using standard protocols. For immunohistochemistry, sections were incubated with anti-4-hydroxy-2-nonenal (4-HNE) antibodies (1:100; MHN-020P, JaICA, Japan). Histological analyses were performed a minimum of five times, using three to five mice per genotype. Histological images were visualized under a microscope (BZ-X710, Keyence, Osaka, Japan). The area of cartilaginous formation in the callus that was stained with safranin O was quantitatively analyzed using ImageJ software (version 1.54; National Institutes of Health). The rates of 4-HNE-positive areas were measured by BZ analyzer software (Keyence).

1.3. Radiological analysis

The left tibias of *Cars2*$^{+/+}$ and *Cars2*$^{\mathrm{AINK}/+}$ mice (n = 4 each) were imaged using a soft X-ray system (M-60; Softex Co., Tokyo).

1.4. Quantitative reverse transcription-polymerase chain reaction (qRT-PCR)

Total RNA was extracted using the Direct-zol RNA kit (#R2062, Zymo Research, Irvine, CA, USA), according to the manufacturer's protocol. Total RNA was reverse-transcribed into cDNA using ReverTraAce qPCR RT Master Mix (#FSQ-201, TOYOBO, Osaka, Japan). qRT-PCR was performed using THUNDERBIRD Next SYBR qPCR Mix (#QPX-201, TOYOBO) and StepOnePlus (Applied Biosystems, Foster City, CA, USA). Relative quantification, based on the standard curve method, was used to compare gene expression levels. Target gene expression levels were normalized using glyceraldehyde-3-phosphate dehydrogenase (Gapdh) as an internal control and following the ΔΔCT method [6]. Three biological and three technical replicates were prepared for each sample (n = 3 per group). The primers used in this study are listed in Supplementary Table 1.

1.5. Bulk RNA-seq

Total RNA samples were prepared as described above and submitted to BGI (BGI, Hong Kong, China) for library preparation and sequencing. Callus of tibias that contain osteochondroprogenitor cells samples were collected from the tibias of *Cars2*$^{+/+}$ and *Cars2*$^{AINK/+}$ mice (n=2 each) at 2 weeks post-fracture. The quality of the purified total RNA was confirmed with an A260/A280 ratio of 1.8–2.0 and an RNA integrity number (RIN) of >7. RNA-seq was performed using a DNBSEQ-G400 (BGI, Shenzhen, China) with 100-bp paired-end reads. Filtered paired-end reads were mapped to the mouse reference genome (GRCm39 GENCODE primary assembly) by HISAT2 (version 2.2.1) and expression levels were quantified by StringTie (version 2.1.7). Statistical analysis was performed by Subio Platform to identify DEGs. Data analysis was conducted using the BGI visualization system (https://www.bgi.com/jp/dr-tom/) and Ingenuity Pathway

Analysis (QIAGEN, Düsseldorf, Germany). Raw and processed data are available in the Gene Expression Omnibus database (www.ncbi.nlm. nih.gov/geo/) under accession number GSE280156.

1.6. Cell cultures

Adipose synovia were resected from knee joints of 12-week-old male *Cars2*$^{+/+}$ mice. The synovia were digested in 2 mg/mL collagenase in high-glucose DMEM (Wako, Osaka, Japan) at 37°C for 1 h and then seeded onto 12-well plates, as previously described [7]. The isolated mouse adipose synovial fibroblasts (ASF) were cultured in high-glucose DMEM with 20% fetal bovine serum (FBS) and 1% penicillin–streptomycin under hypoxic conditions (2% O_2, 5% CO_2). Mice ASF were seeded onto culture dishes and treated with 1 ng/mL interleukin (IL)-1β (Peprotech; Rocky Hill, NJ, USA) and 0, 3, and 30 μM GSSSG for 24 h under hypoxic conditions (2% O_2, 5% CO_2). HEK293T cells were cultured in high glucose DMEM with culture conditions of 10% FBS and 1% penicillin/streptomycin at 37 ºC, 5% CO_2. Mouse chondrogenic ATDC5 cells were cultured in a 1:1 mixture of DMEM and Ham's F12 medium (Thermo Fisher Scientific, Waltham, MA, USA) supplemented with 5% FBS and 1% penicillin-streptomycin at 37 ºC, 5% CO_2.

1.7. Statistical analyses

Data are expressed as the mean ± standard deviation (SD) and analyzed using GraphPad Prism (v.10.2.3; GraphPad Software, San Diego, CA). Statistical significance between two groups was evaluated using a two-tailed Mann–Whitney U test or Student's t-test. For multiple comparisons, one-way ANOVA and Dunnett's post-hoc test were used to

determine significant between-group differences. P-values < 0.05 were considered statistically significant. For the quantitative outcome scoring data, Cohen's d effect size was used to estimate the overall effect size for the OA mouse models, with a power of 0.80 and an alpha level of 0.05 (< 0.2: not clinically relevant; > 0.2: small; > 0.5: moderate; > 0.8: large; > 1.2: very large).

[1] T. Matsunaga, H. Sano, K. Takita, M. Morita, S. Yamanaka, T. Ichikawa, T. Numakura, T. Ida, M. Jung, S. Ogata, S. Yoon, N. Fujino, Y. Kyogoku, Y. Sasaki, A. Koarai, T. Tamada, A. Toyama, T. Nakabayashi, L. Kageyama, S. Kyuwa, K. Inaba, S. Watanabe, P. Nagy, T. Sawa, H. Oshiumi, M. Ichinose, M. Yamada, H. Sugiura, F.Y. Wei, H. Motohashi, T. Akaike, Supersulphides provide airway protection in viral and chronic lung diseases, Nat Commun 14 (2023) 4476. https://doi.org/10.1038/s41467-023-40182-4.

[2] Y. Kitaura, H. Hojo, Y. Komiyama, T. Takato, U.I. Chung, S. Ohba, Gli1 haploinsufficiency leads to decreased bone mass with an uncoupling of bone metabolism in adult mice, PLoS One 9 (2014) e109597. https://doi.org/10.1371/journal.pone.0109597.

[3] S.S. Glasson, M.G. Chambers, W.B. Van Den Berg, C.B. Little, The OARSI histopathology initiative - recommendations for histological assessments of osteoarthritis in the mouse, Osteoarthritis and cartilage 18 Suppl 3 (2010) S17-23. https://doi.org/10.1016/j.joca.2010.05.025.

[4] V. Krenn, L. Morawietz, G.R. Burmester, R.W. Kinne, U. Mueller-Ladner, B. Muller, T. Haupl, Synovitis score: discrimination between chronic low-grade and high-grade synovitis, Histopathology 49 (2006) 358-364. https://doi.org/10.1111/j.1365-2559.2006.02508.x.

[5] T. Ida, T. Sawa, H. Ihara, Y. Tsuchiya, Y. Watanabe, Y. Kumagai, M. Suematsu, H. Motohashi, S. Fujii, T. Matsunaga, M. Yamamoto, K. Ono, N.O. Devarie-Baez, M. Xian, J.M. Fukuto, T. Akaike, Reactive cysteine persulfides and S-polythiolation regulate oxidative stress and redox signaling, Proc Natl Acad Sci U S A 111 (2014) 7606-7611. https://doi.org/10.1073/pnas.1321232111.

[6] K.J. Livak, T.D. Schmittgen, Analysis of relative gene expression data using real-time quantitative PCR and the 2(-Delta Delta C(T)) Method, Methods 25 (2001) 402-408. https://doi.org/10.1006/meth.2001.1262.

[7] H. Sugimoto, Y. Murahashi, R. Chijimatsu, S. Miwa, F. Yano, S. Tanaka, T. Saito, Primary culture of mouse adipose and fibrous synovial fibroblasts under normoxic and hypoxic conditions, Biomed Res 41 (2020) 43-51. https://doi.org/10.2220/biomedres.41.43.

2. Supplemental Figure 1-10

Fig. S1

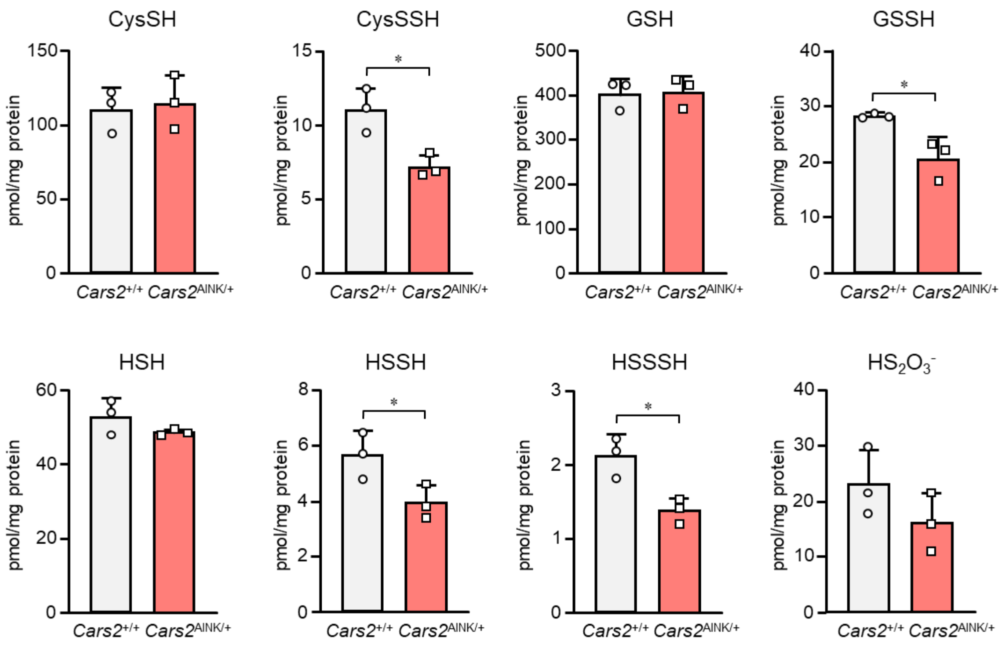

Fig. S1. In vivo formation of supersulfides in *Cars2*$^{+/+}$ and *Cars2*$^{AINK/+}$ mice.

Endogenous production of CysSSH and other related supersulfide metabolites in chondrocytes and chondral tissues obtained from *Cars2*$^{+/+}$ and *Cars2*$^{AINK/+}$ mice littermates were quantified via LC-MS/MS analysis with HPE-IAM labeling. Data are means ± SD (n = 3). *P < 0.05.

Fig. S2

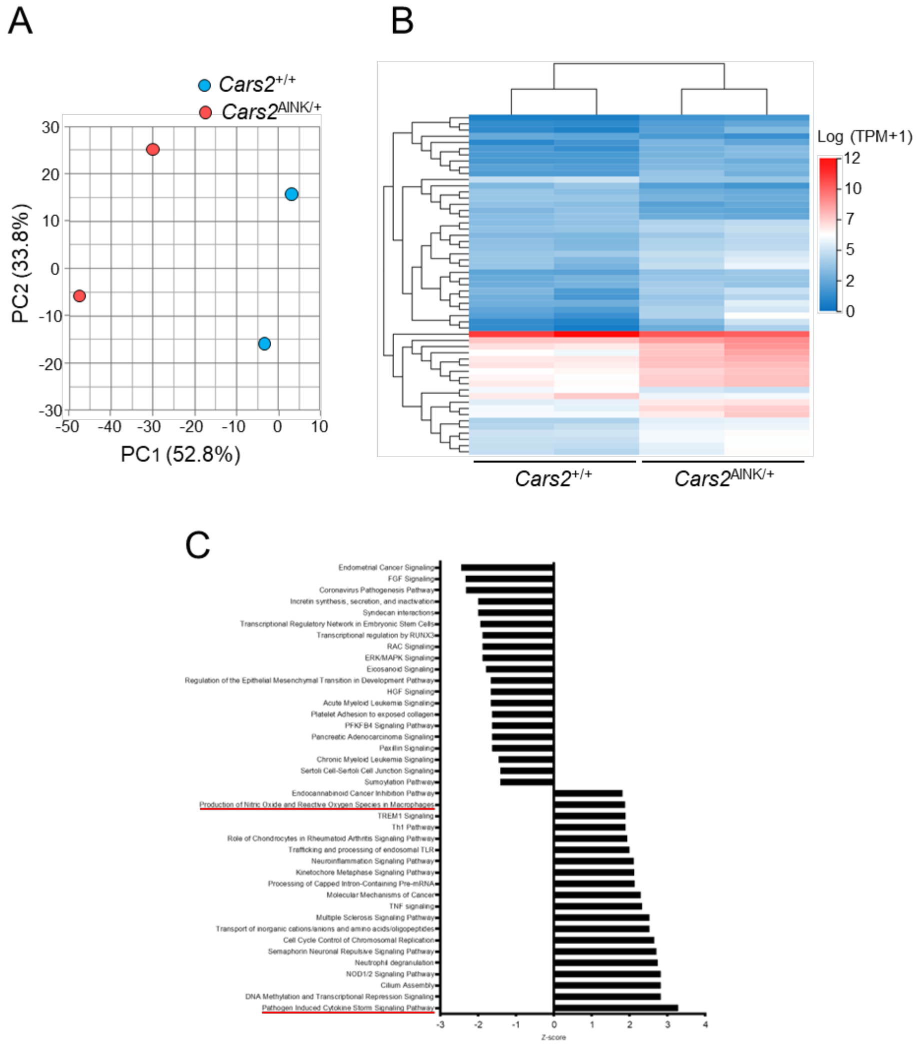

Fig. S2. Comprehensive gene expression in the tibias of *Cars2*$^{+/+}$ and *Cars2*$^{AINK/+}$ mice. (A) Principal component analysis of differentially expressed genes in the tibias of *Cars2*$^{+/+}$ and *Cars2*$^{AINK/+}$ mice. n = 2 mice per group. (B) Heatmaps of gene expression patterns in the tibias of *Cars2*$^{+/+}$ and *Cars2*$^{AINK/+}$ mice. n = 2 mice per group. (C) Ingenuity pathway analysis of the RNA-seq data showing the top 20 upregulated and downregulated pathways in *Cars2*$^{AINK/+}$ mice, as compared with *Cars2*$^{+/+}$ mice.

Fig. S3

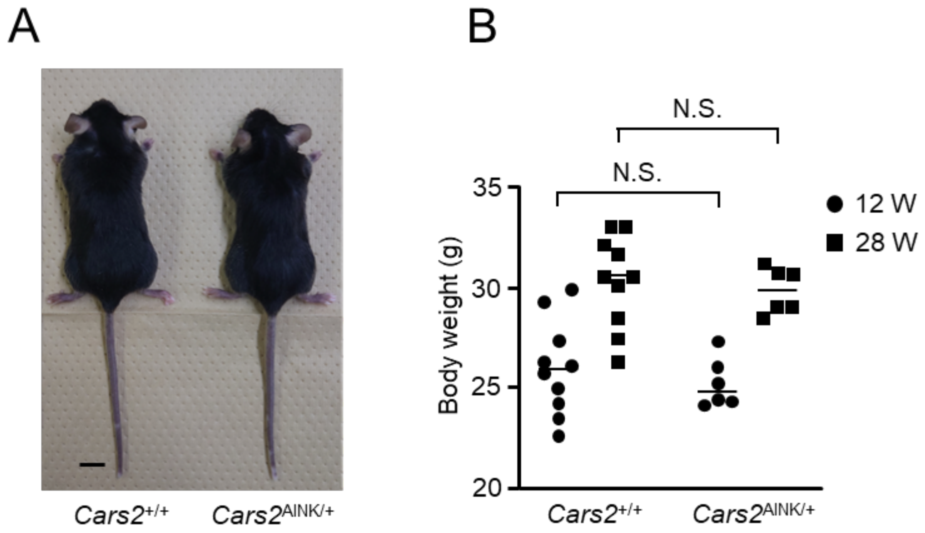

Fig. S3. Gross appearance and body weight of *Cars2*$^{+/+}$ and *Cars2*$^{AINK/+}$ littermates. (A) Gross appearance of *Cars2*$^{+/+}$ and *Cars2*$^{AINK/+}$ littermates at 12-week-old. Scale bars, 1 cm. (B) Body weight of *Cars2*$^{+/+}$ (n = 10) and *Cars2*$^{AINK/+}$ (n = 6) littermates at 12 or 28 week-old. Data are means ± SD. N.S., not significant.

Fig. S4

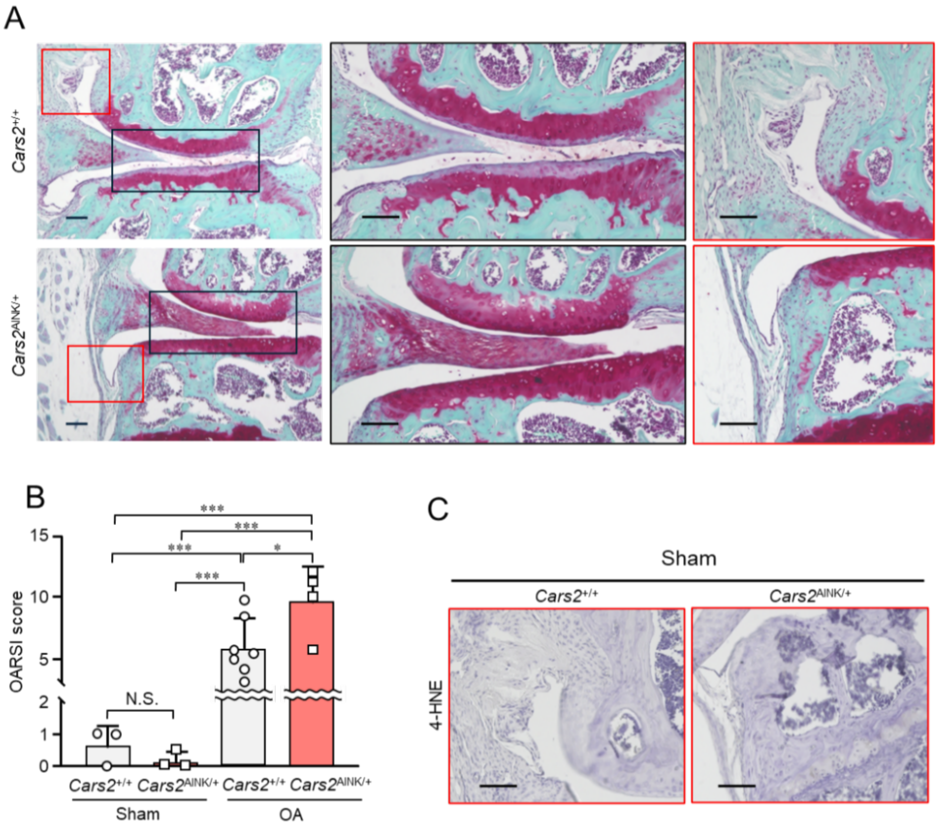

Fig. S4. Development of OA in sham-operated *Cars2*$^{+/+}$ and *Cars2*$^{AINK/+}$ mice.

(A) At 16 weeks post-DMM surgery, sham-operated knee joints were stained with Safranin-O. Boxed areas in the left panels are shown at higher magnification, highlighting articular cartilage (middle panels, outlined in black) and synovial lesions (right panels, outlined in red). Representative images are presented. Scale bars: 100 μm. (B) Quantification of OA development using Osteoarthritis Research Society International (OARSI) histologic scoring (*Cars2*$^{+/+}$ [n = 3] and *Cars2*$^{AINK/+}$ [n = 3] mice for sham, *Cars2*$^{+/+}$ [n = 7] and *Cars2*$^{AINK/+}$ [n = 4] mice for OA model). Data are means ± SD. *P < 0.05, ***P < 0.001. N.S., not significant. (C) Immunohistochemical assessment of 4-hydroxy-2-nonenal (4-HNE) expression in the synovium of knee joints that correspond to the boxed area in (A).

Fig. S5

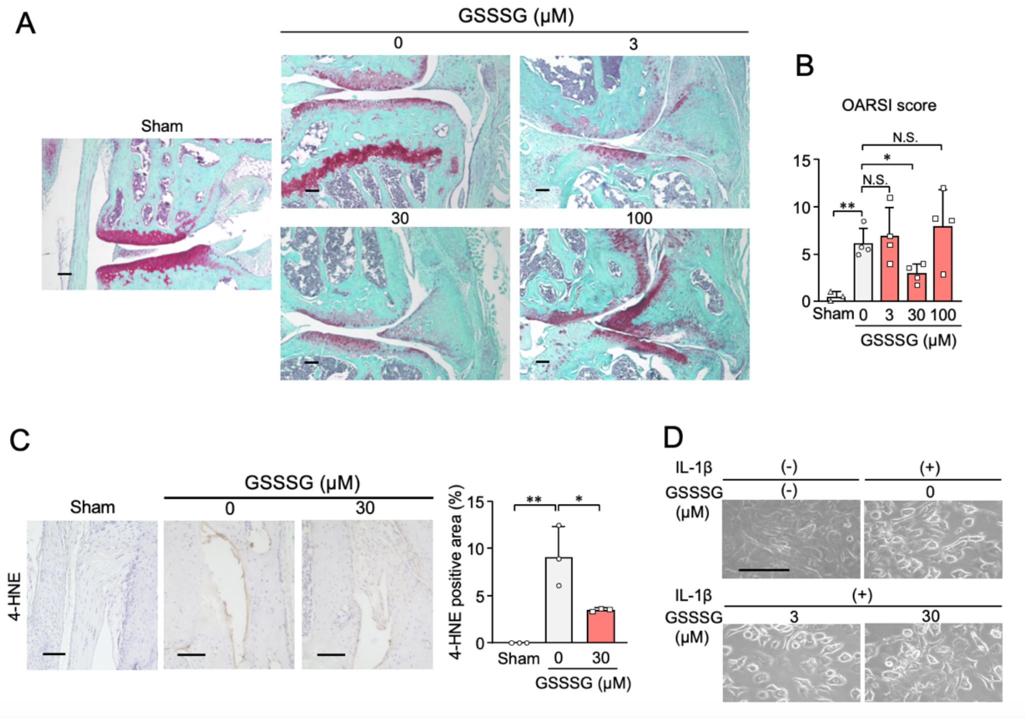

Fig. S5. Effects of intra-articular administration of the supersulfide donor on surgically-induced mouse knee osteoarthritis (OA).

(A) At 16 weeks post-DMM surgery, sham-operated and GSSSG treated (0 to 100 μM), knee joints were stained with safranin-O. Scale bar, 100 μm. (B) Semi-quantification of OA development using OARSI histologic scoring in the GSSSG-treated OA model. Data are means ±SD. *P < 0.05, **P < 0.01, vs. 0 μM; N.S., not significant. Each group (sham-operation, 0, 3, 30, 100 μM treatment) includes 3-4 mice. (C) Immunohistochemical assessment of 4-HNE formation in the synovium of knee joints of sham-operated and GSSSG-treated mice at 16 weeks after OA induction. The right panel indicates the rates of 4-HNE-positive areas. Data are means ±SD. *P < 0.05, **P < 0.01, vs. 0 μM; Student's t-test. (D) Mouse adipose synovial fibroblasts (ASF) cultured under hypoxic conditions (2% O_2, 5% CO_2) and treated with different concentrations of GSSSG, with or without exposure to 1 ng/mL IL-1β. Scale bar, 100 μm.

Fig. S6

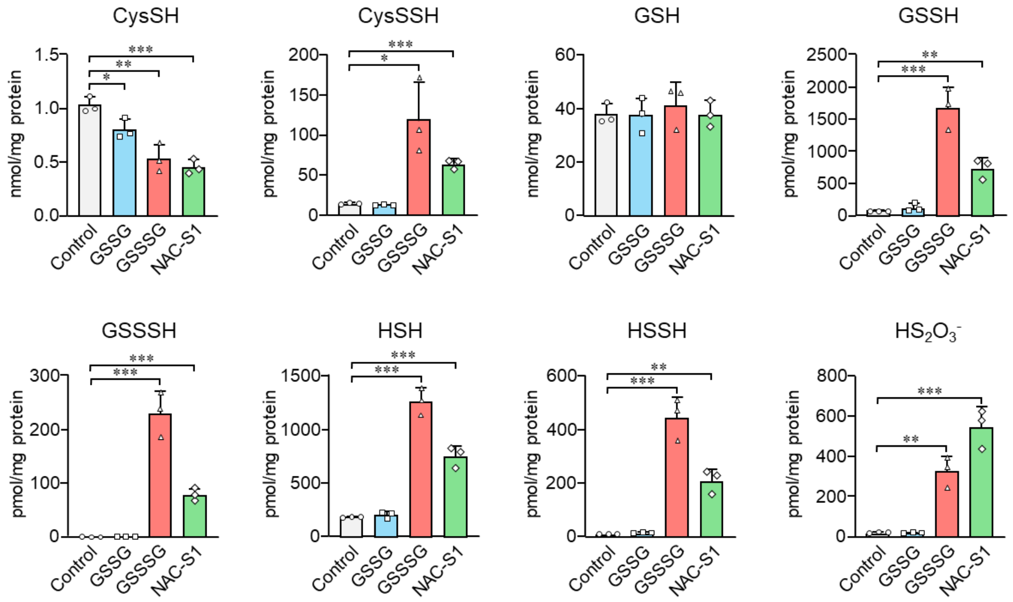

Fig. S6. Cellular uptake analysis in HEK293T cells.

Supersulfide metabolome analysis with HEK293T cells treated with GSSG (200 μM), GSSSG (200 μM), and N-acetylcysteine trisulfide (NAC-S1, 200 μM) for 3 h. The amounts of GSSH in control and 200 μM GSSSG treatment are 71.0 pmol/mg protein (4.01 μM) and 1843 pmol/mg protein (104 μM), respectively (each value of intracellular concentration determined with cell volume is shown in parentheses). Data are means ± SD. (n = 3). *P < 0.05, **P < 0.01, ***P < 0.001.

Fig. S7

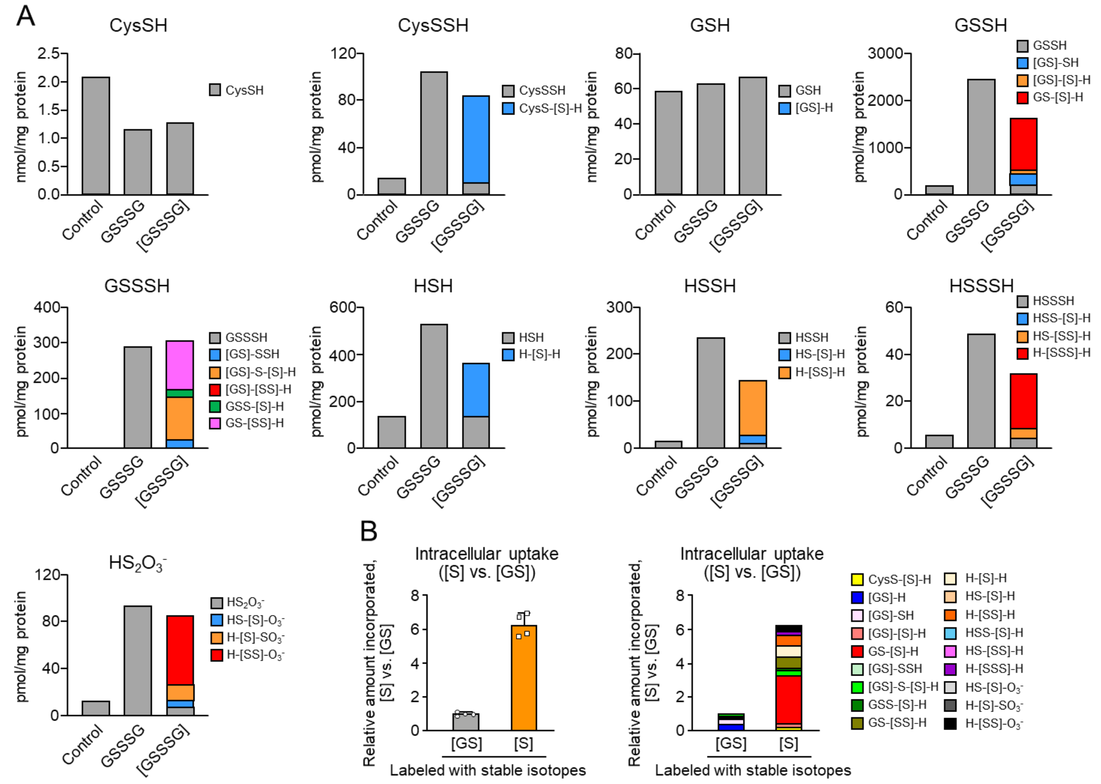

Fig. S7. Cellular uptake analysis of stable isotope-labeled GSSSG in HEK293T cells.

(A) The profile of intracellular uptake of GSSSG with HEK293T cells treated with GSSSG (200 µM) and stable isotope-labeled GSSSG ([GSSSG], 200 µM) for 3 h. Data are presented as means of n = 4. (B) Intracellular uptake of stable isotope-labeled GS ([GS]) and S ([S]) with HEK293T cells treated with stable isotope-labeled GSSSG (200 µM) for 3 h. Left pannel shows the relative ratio ([S] vs. [GS]) of the amount of intracellular [GS] and [S] quantified in (A). Right panel illustrates the relative proportions of each constituent component shown in the left panel.

Fig. S8

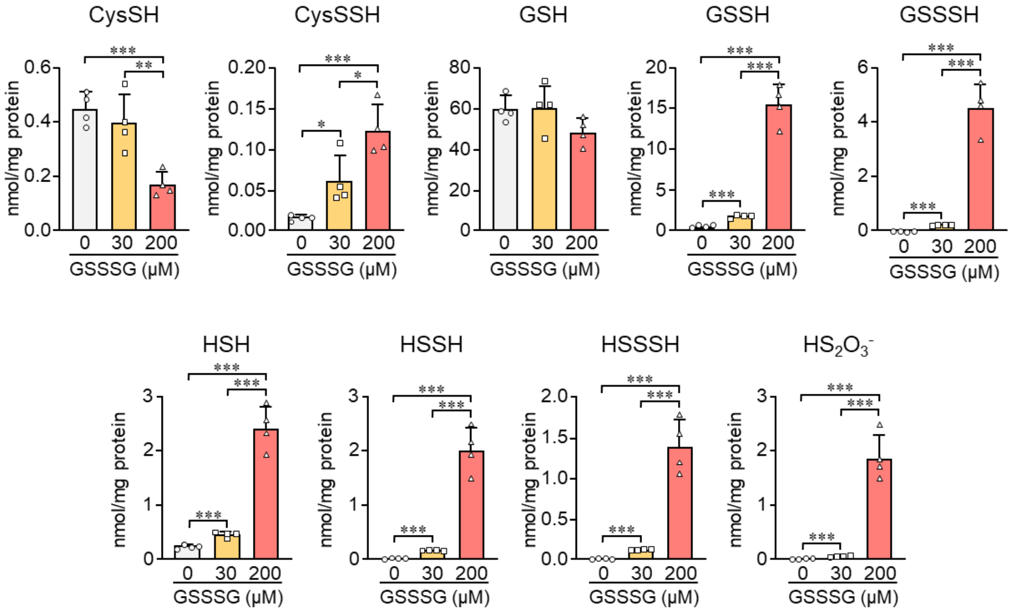

Fig. S8. Cellular uptake analysis in ATDC5 cells.

Supersulfide metabolome analysis with ATDC5 cells treated with various doses of GSSSG (0, 30, 200 μM) for 3 h. The amounts of GSSH in 0 and 200 μM GSSSG treatment are 0.555 nmol/mg protein (44.1 μM) and 15.5 nmol/mg protein (1.23 mM), respectively (each value of intracellular concentration determined with cell volume is shown in parentheses). Data are means ± SD. (n = 4). *P < 0.05, **P < 0.01, ***P < 0.001.

Fig. S9

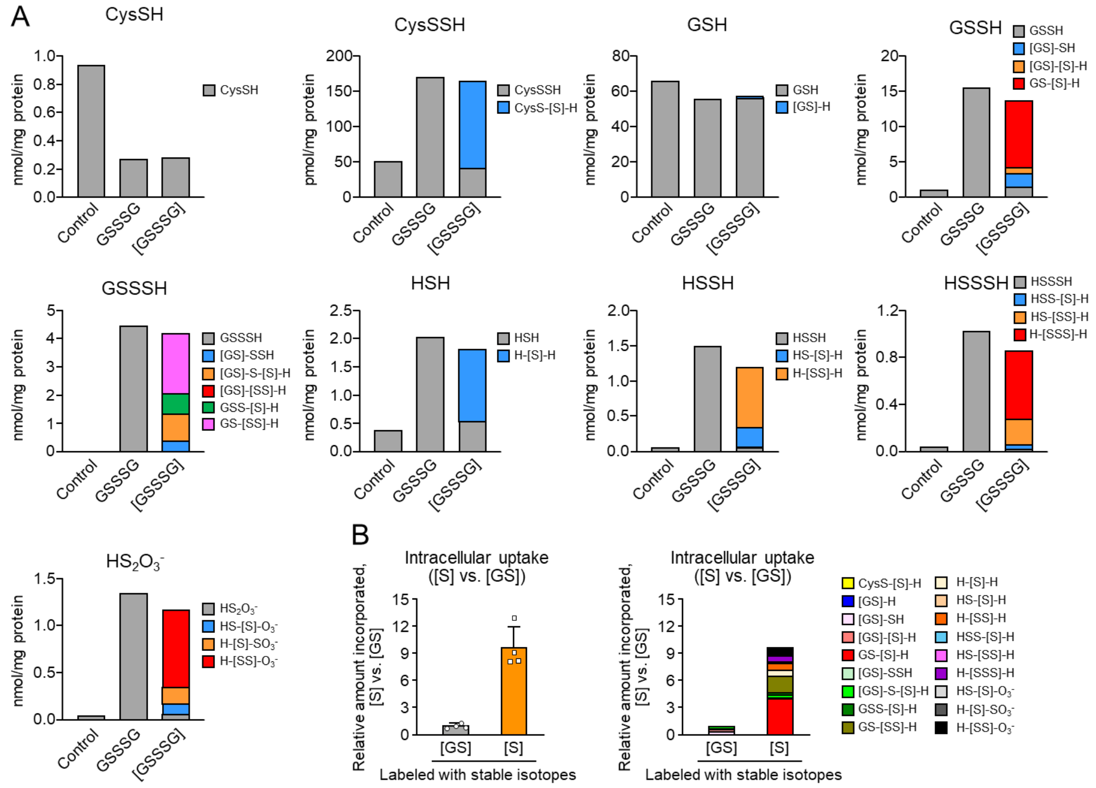

Fig. S9. Cellular uptake analysis of stable isotope-labeled GSSSG in ATDC5 cells. (A) The profile of intracellular uptake of GSSSG with ATDC5 cells treated with GSSSG (200 μM) and stable isotope-labeled GSSSG ([GSSSG], 200 μM) for 3 h. Data are presented as means of n = 4. (B) Intracellular uptake of stable isotope-labeled GS ([GS]) and S ([S]) with ATDC5 cells treated with stable isotope-labeled GSSSG (200 μM) for 3 h. Left pannel shows the relative ratio ([S] vs. [GS]) of the amount of intracellular [GS] and [S] quantified in (A). Right panel illustrates the relative proportions of each constituent component shown in the left panel.

Fig. S10

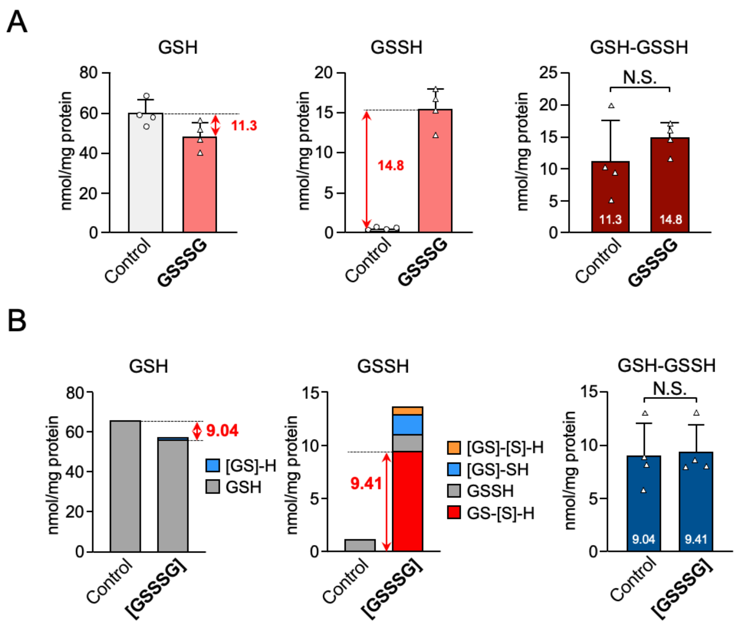

Fig. S10. Stoichiometry of GSH to GSSH conversion in ATDC5 cells after GSSSG treatment.

(A) Difference in intracellular concentrations of GSH and GSSH between GSSSG (200 μM) -treated or -untreated (control) ATDC5 cells. (B) Difference in intracellular concentrations of GSH and GSSH between stable isotope-labeled GSSSG ([GSSSG], 200 μM) -treated or -untreated (control) ATDC5 cells. Data are means ± SD (n = 4). N.S., not significant.

3. Supplemental Table 1-3

Supplemental Table 1

Gene Symbol		Sequence
Col2a1	F	AAGGATGGCTGCACGAAACA
	R	CGGGAGGTCTTCTGTGATCG
Col10a1	F	GCTGAACGGTACCAAACGC
	R	TGCCTTGTTCTCCTCTTACTGG
Bglap	F	AAGCAGGAGGGCAATAAGGT
	R	TTTGTAGGCGGTCTTCAAGC
Sparc	F	CACCTGGACTACATCGGACCAT
	R	CTGCTTCTCAGTGAGGAGGTTG
Il6	F	TACCACTTCACAAGTCGGAGGC
	R	CTGCAAGTGCATCATCGTTGTTC
Il1b	F	TGGACCTTCCAGGATGAGGACA
	R	GTTCATCTCGGAGCCTGTAGTG
CCl2	F	GCTACAAGAGGATCACCAGCAG
	R	GTCTGGACCCATTCCTTCTTGG
Gapdh	F	AGGTCGGTGTGAACGGATTTG
	R	TGTAGACCATGTAGTTGAGGTCA
KIIK	F	GTGTCGAGAAGCCAGAAAAT
	R	AAGGGTCACAAGTACTAGGA

Supplemental Table 2. List of the top 20 z-scores among downregulated Ingenuity Canonical Pathways.

The top 20 z-scores among downregulated pathways	z-score
Endometrial Cancer Signaling	-2.449
FGF Signaling	-2.333
Coronavirus Pathogenesis Pathway	-2.324
Incretin synthesis, secretion, and inactivation	-2
Syndecan interactions	-2
Transcriptional Regulatory Network in Embryonic Stem Cells	-1.941
Transcriptional regulation by RUNX3	-1.89
RAC Signaling	-1.89
ERK/MAPK Signaling	-1.886
Eicosanoid Signaling	-1.8
Regulation of the Epithelial Mesenchymal Transition in Development Pathway	-1.667
HGF Signaling	-1.667
Acute Myeloid Leukemia Signaling	-1.667
Platelet Adhesion to exposed collagen	-1.633
PFKFB4 Signaling Pathway	-1.633
Pancreatic Adenocarcinoma Signaling	-1.633
Paxillin Signaling	-1.633
Chronic Myeloid Leukemia Signaling	-1.46
Sertoli Cell-Sertoli Cell Junction Signaling	-1.414
Sumoylation Pathway	-1.414

Supplemental Table 3. List of top 20 z-score among Ingenuity Canonical Pathways upregulated.

The top 20 z-scores among upregulated pathways	z-score
Pathogen Induced Cytokine Storm Signaling Pathway	3.286
DNA Methylation and Transcriptional Repression Signaling	2.828
Cilium Assembly	2.828
NOD1/2 Signaling Pathway	2.828
Neutrophil degranulation	2.744
Semaphorin Neuronal Repulsive Signaling Pathway	2.714
Cell Cycle Control of Chromosomal Replication	2.646
Transport of inorganic cations/anions and amino acids/oligopeptides	2.53
Multiple Sclerosis Signaling Pathway	2.524
TNF signaling	2.333
Molecular Mechanisms of Cancer	2.292
Processing of Capped Intron-Containing Pre-mRNA	2.132
Kinetochore Metaphase Signaling Pathway	2.121
Neuroinflammation Signaling Pathway	2.117
Trafficking and processing of endosomal TLR	2
Role of Chondrocytes in Rheumatoid Arthritis Signaling Pathway	1.941
Th1 Pathway	1.897
TREM1 Signaling	1.897
Production of Nitric Oxide and Reactive Oxygen Species in Macrophages	1.886
Endocannabinoid Cancer Inhibition Pathway	1.807
